# Management of heart failure with preserved ejection fraction: from neurohormonal antagonists to empagliflozin

**DOI:** 10.1007/s10741-022-10228-8

**Published:** 2022-04-29

**Authors:** Alberto Aimo, Michele Senni, Andrea Barison, Giorgia Panichella, Claudio Passino, Antoni Bayes-Genis, Michele Emdin

**Affiliations:** 1 Institute of Life Sciences, Scuola Superiore Sant’Anna, Pisa, Italy; 2 Cardiology and Cardiovascular Medicine Department, Fondazione Toscana Gabriele Monasterio, Pisa, Italy; 3 Cardiovascular Department & Cardiology Unit, ASST Papa Giovanni XXIII - Bergamo, Bergamo, Italy; 4 ICREC (Heart Failure and Cardiac Regeneration) Research Programme, Health Sciences Research Institute Germans Trias I Pujol (IGTP), Badalona, Spain; 5 Hospital Universitari Germans Trias I Pujol, Badalona (Barcelona), Spain; 6 CIBER Cardiovascular, Instituto de Salud Carlos III, Madrid, Spain

**Keywords:** Heart failure, Preserved ejection fraction, HFpEF, Therapies, Clinical trials

## Abstract

Heart failure with preserved ejection fraction (HFpEF) is a highly prevalent syndrome with multifaceted pathophysiology. All approaches to neurohormonal modulation were shown not to improve survival in HFpEF, despite their well-established efficacy in heart failure with reduced ejection fraction (HFrEF). This might be attributed to suboptimal study design, inadequate diagnostic criteria, or statistical power, but is also likely to reflect a lack of consideration for its clinical heterogeneity. The attention then shifted to the phenotypic heterogeneity of HFpEF, with the ultimate goal of developing therapies tailored to individual patient phenotypes. Recently, the sodium-glucose co-transporter-2 inhibitor (SGLT2i) empagliflozin has been found to reduce the combined risk of cardiovascular death or hospitalization for HF in patients with HFpEF, a result driven by a reduction in HF hospitalizations. This paper recapitulates the journey from the failure of trials on neurohormonal antagonists to the attempts of personalized approaches and the new perspectives of SGLT2i therapy for HFpEF.

Heart failure with preserved ejection fraction (HFpEF) is a syndrome with complex and multifaceted pathophysiology, which includes a crucial role of comorbidities. The apparent impossibility to find a “one-size-fits-all” treatment for HFpEF prompted a dissection of the phenotypic spectrum of this condition, with the ultimate goal of tailoring treatment on individual phenotypes. Recently, a standardized therapy was found to confer a prognostic benefit in HFpEF. Indeed, the sodium-glucose co-transporter-2 inhibitor (SGLT2i) empagliflozin reduced the combined risk of cardiovascular death or hospitalization for HF in patients with HFpEF, a result driven by a reduction in HF hospitalizations [1]. This paper recapitulates the journey from the failure of trials on neurohormonal antagonists to the attempts of personalized approaches and the new perspectives of SGLT2i therapy for HFpEF. Trials will be evaluated regardless of their specific definition of HFpEF, which often included patients with EF values lower than 50%.

## Guideline recommendations

The 2021 European Society of Cardiology (ESC) guidelines retained the classification of HF into 3 categories: HF with reduced EF (HFrEF; EF ≤ 40%), HF with mildly reduced EF (HFmrEF; EF 41–49%), and HFpEF (EF ≥ 50%) [2]. All classes of therapies for neurohormonal antagonism (angiotensin-converting enzyme inhibitors [ACEi], angiotensin receptor blockers [ARBs], beta-blockers, mineralocorticoid receptor antagonists [MRAs], and sacubitril/valsartan) may be considered in patients with HFmrEF, based on consensus opinion (class IIb, level of evidence C) [2]. According to both 2021 ESC and 2017 American College of Cardiology/American Heart Association/Heart Failure Association of America (ACC/AHA/HFSA) guidelines [3], comorbidities should be searched and treated in patients with HFpEF, and diuretics should be used to relieve congestion (class I, level of evidence C recommendations) [2]. ACC/AHA/HFSA guidelines add that MRA might be considered in patients with EF ≥ 45%, elevated B-type natriuretic peptide (BNP), or HF hospitalization within 1 year, no stage 4 or 5 chronic kidney disease or hyperkalemia [3], reflecting the positive results of the Treatment of Preserved Cardiac Function HF with an Aldosterone Antagonist (TOPCAT) trial in the USA (see below).

## Therapies for neurohormonal antagonism

Drugs counteracting neurohormonal activation target a key pathophysiological mechanism of HFrEF and have greatly improved the prognosis of this condition. When the same drugs have been evaluated in HFpEF, clinical trials have consistently produced disappointing results (Table 1).

**Table 1:** Clinical trials on neurohormonal drugs to treat heart failure with preserved ejection fraction (HFpEF)

**Study, ref**	**Drug**	**Patient number**	**Mean age (years)**	**Women (%)**	**LVEF**	**Endpoints**	**HR**	***p*-value**	**Follow-up**
					**criterion**		**(95% CI)**		
***Beta-blockers***									
CIBIS-ELD (HFpEF subgroup) [5]	Bisoprolol or carvedilol (no placebo)	250	73	66	> 45%	LVEF change (%)	0.4 (− 0.6 to 1.4)	0.47	12 weeks
						LV diastolic dysfunction change	− 0.04 (− 0.10 to 0.03)	0.28	
						NYHA class change	− 0.18 (− 0.25 to − 0.11)	** < **0.001	
						6MWD change (m)	4 (− 8 to 16)	0.52	
SENIORS (HFpEF subgroup) [71]	Nebivolol	752	76	38	> 35%	All-cause death + CV hospitalization	0.82 (0.63–1.05)	0.2	21 months
Swedish HF registry [6]	Beta-blockers (retrospective)	8244	78	46	> 40%	All-cause death	0.93 (0.83–0.99)	0.04	24 months
						All-cause death + HF hospitalization	0.98 (0.92–1.04)	0.46	
***ACEi/ARB***									
CHARM-Preserved [8]	Candesartan	3023	67	40	> 40%	CV death + HF hospitalization	0.86 (0.74–1.00)	0.05	37 months
						CV death	0.95 (0.76–1.18)	0.64	
						HF hospitalization	0.84 (0.70–1.00)	0.047	
I-Preserve [9]	Irbesartan	4128	72	60	> 40%	All-cause death + HF hospitalization	0.95 (0.86–1.05)	0.35	50 months
						CV death	0.98 (0.63–1.53)	0.93	
						HF hospitalization	0.86 (0.61–1.20)	0.38	
PEP-CHF [7]	Perindopril	850	75	56	> 45%	All-cause death + HF hospitalization	0.92 (0.70–1.21)	0.55	26 months
						CV death	0.98 (0.63–1.53)	0.93	
						HF hospitalization	0.86 (0.61–1.20)	0.38	
***MRA***									
TOPCAT [11]	Spironolactone	3445	69	52	≥ 45%	CV death + HF hospitalization + aborted cardiac arrest	0.89 (0.77–1.04)	0.14	27 months
						CV death			
						HF hospitalization	0.90 (0.73–1.12)	0.35	
						Aborted cardiac arrest	0.83 (0.69–0.99)	0.04	
							0.60 (0.14–2.50)	0.48	
Aldo-DHF [10]	Spironolactone	422	67	52	> 50%	LV diastolic function (*E*/*e*′)	− 1.5 (− 2 to − 0.9)	** < **0.001	12 months
						Peak O_2_ consumption (ml/Kg/min)	0.1 (− 0.6 to 0.8)	0.81	
						LV mass index (g/m^2^)	− 6 (− 10 to − 1)	0.009	
***ARNI***									
PARAMOUNT [13]	Sacubitril/valsartan	301	71	56	≥ 45%	NT-proBNP	0.77 (0.64–0.92)	0.005	36 months
						LA volume (ml)		0.003	
						LV diastolic function (*E*/*e*′)		0.42	
PARAGON-HF [14]	Sacubitril/valsartan	4796	73	52	≥ 45%	CV death + HF hospitalization	0.87 (0.75 − 1.01)	0.06	35 months
						CV death	0.95 (0.79 − 1.16)	NS	
						HF hospitalization	0.85 (0.72 − 1.00)	0.05	

Beta-blockers slow heart rate, reduce myocardial contractility, and increase the time for ventricular filling during diastole. The Study of Effects of Nebivolol Intervention on Outcomes and Rehospitalisation in Seniors with HF (SENIORS) trial randomized patients > 75 years with either EF < 35% or a HF hospitalization within the previous 6 months to nebivolol or placebo. Among patients with EF > 35% (*n* = 752, 35%), nebivolol therapy did not reduce all-cause deaths or HF hospitalizations and did not improve systolic or diastolic function [4]. In the Cardiac Insufficiency Bisoprolol Study in Elderly (CIBIS-ELD) trial, bisoprolol did not improve clinical parameters and LV function in the subset with HFpEF (EF > 45%; *n* = 250, 29%) [5]. In the Swedish Heart Failure Registry, 8244 patients with HFpEF (defined as EF > 40%) were matched 2:1 based on age and beta-blocker use [6]. Over a median follow-up time of 755 days, beta-blocker therapy did not impact the composite of all-cause death or HF hospitalization [6].

Angiotensin-II promotes myocardial hypertrophy and fibrosis, two cardinal features of HFpEF. In the Perindopril in Elderly People with chronic HF (PEP-CHF) trial, 850 patients ≥ 70 years, with EF > 45% and diastolic dysfunction (DD) were randomized to perindopril or placebo [7]. Over the first year of follow-up, patients on perindopril had fewer HF hospitalizations, improved New York Heart Association (NYHA) functional class, and 6-min walking distance (6MWD), but the effect on HF hospitalization was lost over the entire follow-up (median 2.1 years) [7]. In the Candesartan in patients with chronic HF and preserved left-ventricular ejection fraction (CHARM-Preserved) trial, enrolling 3023 patients with EF > 40%, a borderline effect of candesartan on the primary endpoint (cardiovascular death or HF hospitalization) was observed over a median 37-month follow-up (*p* = 0.051) [8]. The study was limited by the low EF cutoff for HFpEF and a high rate of candesartan discontinuation. Furthermore, there was a limited characterization of diastolic function in the overall population, with only 44% of patients showing moderate or severe DD [8]. The irbesartan in patients with HF and preserved ejection fraction (I-Preserve) trial randomized 4128 HFpEF patients (EF > 45%) to irbesartan or placebo [9]. During a mean 49.5-month follow-up, no significant differences were observed in all-cause mortality or HF hospitalization [9]. The high rate of irbesartan discontinuation (34% by the end of the study) was a potential reason for these neutral results. Furthermore, frequent use of at least another drug acting on the renin–angiotensin–aldosterone axis (beta-blockers, ACEi, or spironolactone) in both study arms might have reduced the additive benefit from irbesartan [9].

Aldosterone promotes the development of myocardial hypertrophy and fibrosis. In the Aldosterone Receptor Blockade in Diastolic HF (Aldo-DHF) trial, 422 patients with HFpEF (EF > 50%) were randomized to spironolactone or placebo; 1 year of spironolactone treatment was associated with reduced *E*/*e*′ ratio, without significant changes in maximal exercise capacity, patient symptoms or quality of life (QoL) [10]. The TOPCAT trial assessed whether these effects of MRAs translate into a prognostic benefit [11]. Among the inclusion criteria, there were EF ≥ 45%, HF hospitalization within 12 months, or elevated natriuretic peptides (NPs) within 60 days. A total of 3445 patients from 6 countries (USA, Argentina, Brazil, Canada, Russia, and Georgia) were randomized to spironolactone or placebo [11]. Over a mean follow-up of 3.3 years, primary endpoint incidence (a composite of cardiovascular death, aborted cardiac arrest, or HF hospitalization) was 5.9 per 100 person-years in the spironolactone group and 6.6 per 100 person-years in the placebo group (hazard ratio, HR 0.89 [95% confidence interval 0.77–1.04], *p* = 0.14) [11]. In a post hoc analysis, a significant benefit from spironolactone was found in patients enrolled in North and South Americas (HR 0.82 [0.69–0.98], *p* = 0.026), who had an around fourfold higher event rate than those from Russia and Georgia. The latter were younger, had less atrial fibrillation and diabetes mellitus, and were enrolled much more often because of a prior history of HF hospitalization [12].

Sacubitril/valsartan combines the inhibitory action on the renin–angiotensin–aldosterone system with the blockade of neprilysin, the primary enzyme that degrades BNP. The phase 2 Prospective Comparison of ARNI with ARB on Examination of Heart Failure with Preserved Ejection Fraction (PARAMOUNT) trial enrolled 301 patients with HFpEF (EF ≥ 45%, including 75% with EF ≥ 50%), randomized to sacubitril/valsartan 50 mg twice daily (titrated to 200 mg twice daily) or valsartan 40 mg twice daily (titrated to 160 mg twice daily) for 12 weeks (with a 24-week extension period). Sacubitril/valsartan caused a more prominent reduction in N-terminal fraction of pro-BNP (NT-proBNP) and left atrial dimensions than valsartan alone [13]. In the Efficacy and Safety of LCZ696 Compared to Valsartan, on Morbidity and Mortality in Heart Failure Patients With Preserved Ejection Fraction (PARAGON-HF) trial, sacubitril/valsartan (target dose 97/103 mg twice daily) did not reduce the incidence of the primary composite outcome of cardiovascular death or HF hospitalization compared to valsartan (target dose 160 mg twice daily) (HR 0.87 [0.75–1.01], *p* = 0.06) in patients with EF ≥ 45%, NYHA class II to IV, and elevated NPs. Sacubitril/valsartan reduced the primary endpoint in patients with EF < median (57%), patients with reduced glomerular filtration rate, and women [14].

The reason why HFpEF trials failed systematically is unclear, but we may think of several possible reasons, not mutually exclusive: flaws in study design (such as inadequate diagnostic criteria or low statistical power), lower importance of neurohormonal mechanisms in HFpEF than in other forms of HF, and the wide heterogeneity of HFpEF phenotypes [15–17].

## Patient phenotyping to guide treatment

**Fig. 1:**
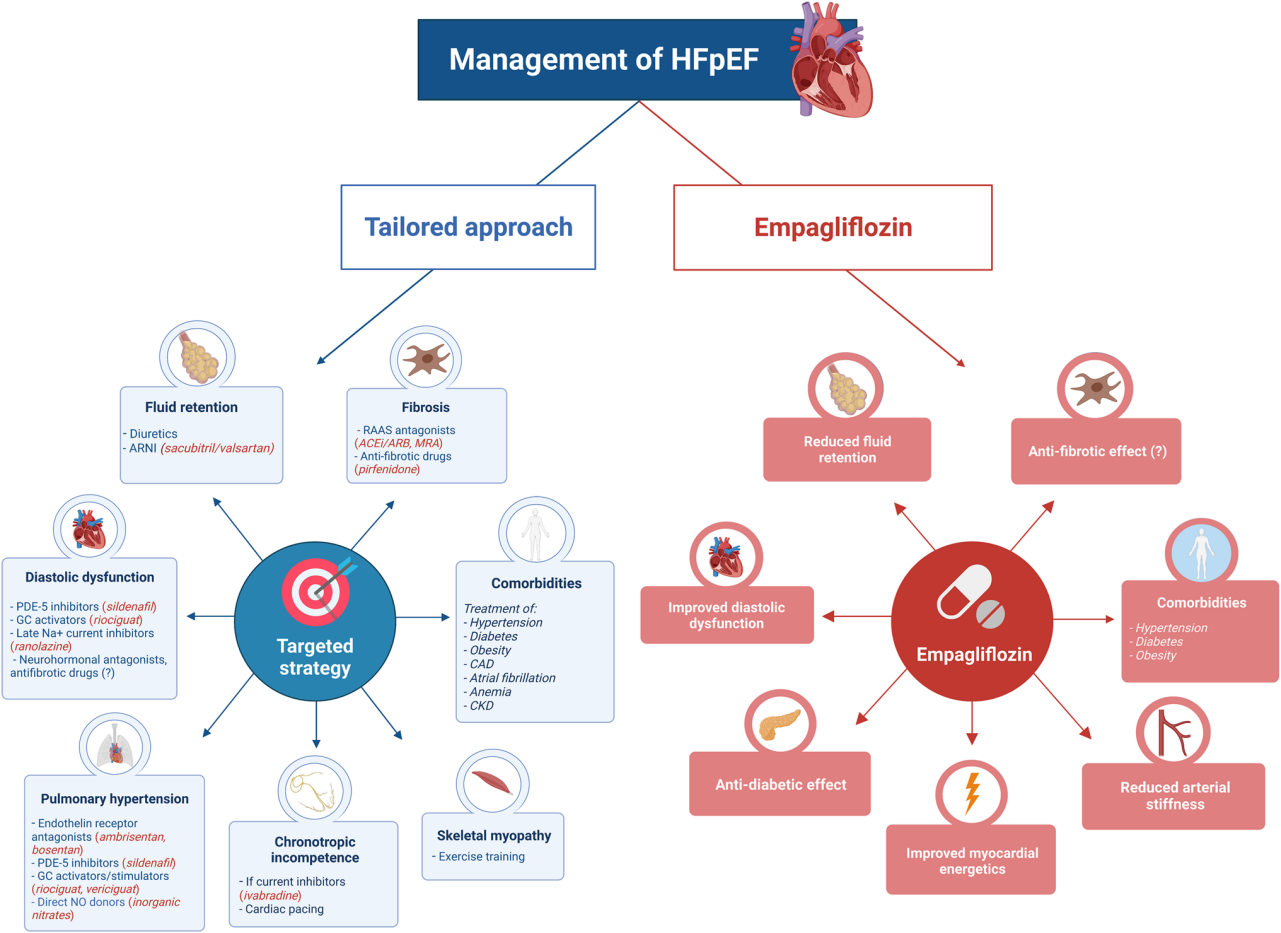
Central illustration. Management of HFpEF. A therapeutic strategy targeting the individual phenotypes of HFpEF patients has been proposed. This approach may implement a standardized treatment represented by empagliflozin and possibly other sodium-glucose co-transporter-2 inhibitors (SGLT2i) such as dapagliflozin. Some of the proposed mechanisms of cardiac protection by SGLT2i are reported in the figure. ACEi/ARB, angiotensin-converting enzyme inhibitors/angiotensin receptor blockers; ARNI, angiotensin receptor/neprilysin inhibitor; CAD, coronary artery disease; CKD, chronic kidney disease; GC, guanylate cyclase; HFpEF, heart failure with preserved ejection fraction; MRA, mineralocorticoid receptor antagonists; NO, nitric oxide; PDE-5, phosphodiesterase-5; RAAS, renin–angiotensin–aldosterone system

The phenotypic heterogeneity of HFpEF has attracted much attention with the ultimate goal of a tailored treatment. Researchers tried to enucleate some features of HFpEF (such as fluid retention or fibrosis) that, when particularly pronounced in individual patients, may predict a better response to specific treatments (for example, diuretics or anti-fibrotic drugs) [15] (Fig. 1).

### Diastolic dysfunction

DD is the hallmark of HFpEF and is produced by abnormalities in both cardiomyocytes and the extracellular matrix. Ranolazine limits increase in diastolic tension, mainly by inhibiting late-sodium current, thus preventing sodium overload and, as a result, calcium accumulation in the cell [18]. The Ranolazine for the Treatment of Diastolic Heart Failure (RALI-DHF) trial compared the effects of ranolazine and placebo on hemodynamic function, indicators of DD, and biomarkers in 20 patients with HFpEF (EF ≥ 45%) and DD. After 30 min of infusion, significant decreases from baseline were observed in left ventricular (LV) end-diastolic pressure and pulmonary capillary wedge pressure in the ranolazine group. After 14 days of treatment, however, no significant changes were observed in echocardiographic or cardiopulmonary exercise test parameters, nor in NT-proBNP levels [19]. A meta-analysis of 4 studies on MRA reported a significant reduction in mean E/e′ ratio, but no significant changes in deceleration time and E/A ratio in patients with HFpEF (EF ≥ 50%, *n* = 3; EF 45–49%, *n* = 1) receiving spironolactone or eplerenone [20]. In summary, several treatments have shown limited effects on diastolic function, which are likely too small to translate into any prognostic benefit.

### Fluid retention

Diuretics lower LV pressures and reduce lung congestion but are often insufficient to control symptoms, do not improve outcomes, may cause renal dysfunction and hypotension; electrolyte and fluid imbalances due to diuretic utilization include hypokalemia, hyponatremia, metabolic acidosis, and hypomagnesemia. Guidelines recommend the use of diuretics to improve symptoms and signs associated with congestion but do not provide any guidance on which diuretic classes, combination, and titration scheme should be used. Loop diuretics, thiazides, and potassium-sparing diuretics are the most commonly used diuretic in HFpEF, either alone or in combination, with slightly different pharmacokinetics, pharmacodynamics, and side effects [21]. A few previous clinical studies have suggested that even loop diuretics are not necessarily a homogeneous class and that furosemide is not necessarily preferable as compared with other loop diuretics [22, 23]. Overall, diuretics have not been demonstrated to improve long-term prognosis, also because of ethical difficulty in designing randomized and prospective clinical trials. Moreover, HFpEF patients might present a different response to diuretic therapy compared to HFrEF patients, so future trials should be designed separately for HFrEF and HFpEF patients [24]. In a retrospective study on 445 discharged patients with HFpEF and a LVEF ≥ 50%, loop diuretics were the only therapy to be associated with a lower hospital readmission risk in the 30 days following an index hospitalization [25], but long-term data were not available.

Recently, two completely different pharmacological therapies with pleiotropic (diuretic and non-diuretic) cardiovascular effects have entered the pharmacological armamentarium. On the one hand, sacubitril/valsartan, which promotes diuresis and natriuresis by increasing BNP, has been shown to improve prognosis in women and patients with an EF of 45–57% [26] (see above). On the other hand, empagliflozin, which promotes glycosuria and natriuresis, has shown a prognostic benefit across a wide range of HFpEF patients (see below). Volume overload in HFpEF is particularly sensitive to renal dysfunction in a vicious cardio-renal cycle and, likely a modifiable risk factor, should be promptly addressed with diuretic drugs to decrease the incidence and morbidity of HFpEF [27]. Nevertheless, the choice of the most effective and least nephrotoxic diuretic therapy should be preferred, and this might explain the clinical and prognostic benefit of these novel “diuretic” therapies with plenty of pleiotropic cardiorenal effects compared to more traditional diuretic classes.

### Fibrosis

Myocardial fibrosis is common in patients with HFpEF, usually occurring in the context of cardiac hypertrophy and microvascular rarefaction [28]. Both MRA [20] and sacubitril/valsartan [29] have been reported to reduce biomarkers of collagen turnover. The clinical relevance of these effects is unclear.

Myocardial fibrosis shares some pathophysiological mechanisms with other fibrotic diseases, such as idiopathic pulmonary fibrosis (IPF) [30]. Pirfenidone, an anti-fibrotic drug approved for clinical use in IPF, could therefore play a role in HFpEF treatment. The Pirfenidone in Patients With Heart Failure and Preserved Left Ventricular Ejection Fraction (PIROUETTE) trial was a randomized, double-blind, placebo-controlled phase 2 trial evaluating the safety and efficacy of 52 weeks of treatment with pirfenidone in 94 patients with chronic HFpEF and myocardial fibrosis (defined as extracellular volume [ECV] ≥ 27%). Pirfenidone reduced ECV on repeated cardiac magnetic resonance scans (between-group difference, − 1.21% [− 2.12 to − 0.31]; *p* = 0.009). Pirfenidone was associated with a reduction in log NT-proBNP compared to placebo (*p* = 0.02), with no significant differences in diastolic function, 6MWD, and Kansas City Cardiomyopathy Questionnaire (KCCQ) summary score [30].

### Pulmonary hypertension

About 80% of patients with HFpEF have pulmonary hypertension (PH), defined as mean pulmonary artery pressure (PAP) ≥ 25 mmHg [31]. The Safety and Efficacy Trial to Treat Diastolic Heart Failure Using Ambrisentan (NCT00840463) was terminated in 2015 after only 4 patients were randomized. The Safety and Efficacy of Bosentan in Patients With Diastolic Heart Failure and Secondary Pulmonary Hypertension (BADDHY) trial evaluated the efficacy of bosentan on 6MWD, QoL, echocardiographic, and laboratory parameters; this study was stopped after an interim analysis showing an improvement of 6MWD and systolic PAP in the placebo group [32].

Phosphodiesterase-5 (PDE-5) inhibitors induce vasodilation by the accumulation of intracellular cyclic guanosine monophosphate (a mediator of the response to nitric oxide). In a study, 44 patients with HFpEF (EF ≥ 50%) and systolic PAP > 40 mmHg were randomized to placebo or sildenafil 50 mg three times daily for 12 months. Sildenafil reduced mean PAP, indices of right ventricular function, and right atrial pressure at 6 and 12 months [33]. PDE-5 inhibition did not prove effective in the RELAX study, possibly because of lower sildenafil doses, shorter follow-up (12 weeks), and HFpEF patient inclusion regardless of PAP values [34].

Increased cyclic guanosine monophosphate can also be achieved through guanylate cyclase stimulators, riociguat, and vericiguat. A preliminary analysis of phase 2b DYNAMIC trial showed an improvement of cardiac output and pulmonary vascular resistance on riociguat therapy [35]. The VITALITY-HFpEF trial randomized 789 patients with HFpEF (EF ≥ 45%) and NYHA class II-III symptoms, within 6 months of a recent decompensation and with elevated NPs to vericiguat or placebo. Vericiguat at either 10 mg or 15 mg per day failed to improve the physical limitation score of the KCCQ [36].

Direct nitric oxide donors, like organic nitrates, may reduce pulmonary capillary wedge pressure. However, in the Inorganic Nitrite Delivery to Improve Exercise Capacity in Heart Failure with Preserved Ejection Fraction (INDIE-HFpEF) trial, 4-week administration of inhaled inorganic nitrite did not increase exercise capacity, KCCQ score, NYHA class, diastolic function, and NT-proBNP levels [37]. The Effect of KNO3 Compared to KCl on Oxygen UpTake in Heart Failure With Preserved Ejection Fraction (KNO3CK OUT HFpEF) is seeking to assess if potassium nitrate can improve exercise capacity in HFpEF patients (NCT02840799).

### Chronotropic incompetence

Chronotropic incompetence (ChI), defined as the inability to adequately increase heart rate during exercise, is common in HFpEF and may be further worsened by beta-blockers [38]. The Preserve-HR study is an ongoing trial assessing beta-blocker withdrawal in HFpEF patients with ChI (NCT03871803). The Rate-Adaptive Atrial Pacing in Diastolic Heart Failure (RAPID-HF) is exploring the response to dual-chamber pacing in patients with HFpEF and ChI (NCT02145351).

### Skeletal myopathy

HFpEF patients show skeletal muscle abnormalities, such as reduced mass, altered composition with increased intramuscular fat, decreased capillary density, and impaired oxidative metabolism, which reduce exercise tolerance [39]. Importantly, previous studies demonstrated that these abnormalities are not explained by deconditioning or to reduced cardiac output but rather intrinsic to the HFpEF syndrome, likely triggered by circulating and neuroendocrine factors [40]. Exercise training is a safe and effective intervention to improve peak oxygen consumption (VO_2_) and QoL in HFpEF, although it does not significantly affect resting diastolic or systolic function [41]. A novel pharmacological agent (neladenoson bialanate, a partial adenosine A1 receptor agonist) has been specifically designed to target skeletal muscle and myocardial mitochondrial dysfunction but has failed to produce a clinically significant benefit on exercise tolerance in 305 HFpEF patients compared to placebo [42]. On the other hand, metabolic effects of gliflozins include improved insulin sensitivity and fatty acid oxidation in the skeletal muscle, which might at least partly explain their beneficial effect on exercise tolerance HFpEF patients (see below).

### Comorbidities

Hypertension is the most prevalent comorbidity in HFpEF and causes increased afterload, oxidative stress, and vascular inflammation. Thiazide diuretics and drugs acting on the angiotensin/aldosterone pathway (including sacubitril/valsartan) [43] seem effective as antihypertensive agents.

HFpEF is often associated with type II diabetes. Hyperglycemia induces the formation of advanced glycation end-products (AGEs), whose deposition in the heart increases collagen production and cross-linking, and activation of AGE receptors; the latter impairs calcium homeostasis, induce profibrotic signaling and endothelial dysfunction, reduce nitric oxide availability, and promote oxidative stress and inflammation [44]. Alagebrium chloride breaks AGE crosslinks; in a small study, it improved diastolic function and QoL in elderly patients with HFpEF [45]. In the PROLOGUE trial, sitagliptin (a dipeptidyl peptidase 4 inhibitor) did not reduce NT-proBNP nor relieved systolic dysfunction on top of conventional antidiabetic treatment over 24 months [46]. The groundbreaking results of empagliflozin are discussed below.

Obesity induces hemodynamic and laboratory changes that are associated with functional and structural cardiac remodeling, ultimately leading to HFpEF [47]. Caloric restriction and aerobic exercise can improve peak VO2, but they seem to have no effect on QoL [47]. The phase 3 Semaglutide Treatment Effect in People with obesity and HFpEF (STEP-HFpEF) trial will assess the effect of glucagon-like peptide 1 semaglutide 2.4 mg once/week on symptoms, body weight, and functional capacity in obese HFpEF patients (NCT04788511).

Anemia is an independent predictor of worse outcomes in HFpEF [48]. Treatment with epoetin alfa for 24 weeks did not modify 6MWD, LV mass, or function in 56 patients with HFpEF (EF ≥ 40%) over 6 months [48]. The Effect of IV Iron in Patients With Heart Failure With Preserved Ejection Fraction (FAIR-HFpEF; NCT03074591) trial will assess whether ferric carboxymaltose improves symptoms and exercise capacity in patients with EF ≥ 45% and iron deficiency, with or without anemia; the Effects of Iron Therapy in Heart Failure With Preserved Ejection Fraction and Iron Deficiency (PREFER*-*HF; NCT03833336) trial will compare the effects of intravenous or oral iron in a similar population.

## Empagliflozin

### Cardiac protective effects

SGLT2i were developed as oral antidiabetic drugs that inhibit glucose reabsorption in the proximal renal tubules, thus lowering serum glucose levels in an insulin-independent way [49]. SGLT2i causes a prominent reduction in HF hospitalization rates in diabetic patients [50]. The mechanisms of cardiac protection by SGLT2i have not been fully clarified, although several hypotheses have been proposed. Empagliflozin increases natriuresis and osmotic diuresis, which results in lowering cardiac preload and afterload [49]. Empagliflozin reduces blood pressure without a compensatory sympathetic stimulation [49], then without increases in heart rate, and it also reduces arterial stiffness and vascular resistance [51]. Several metabolic effects have also been observed: empagliflozin is associated with decreased body weight, fat mass [52], and serum uric acid levels [53], without causing hypoglycemia [54]. Furthermore, empagliflozin fosters ketogenesis and causes a shift from glucose to fat oxidation [55]. Ketone bodies may provide an additional source of energy for the failing heart, improve endothelial and mitochondrial function, as well as mitigate inflammation, oxidative stress, and cardiac remodelling [56]. However, the cardioprotective mechanisms of empagliflozin seem to be not limited to its hemodynamic and metabolic benefits, but it also directly affects heart structure, function, and bioenergetics. Empagliflozin is associated with attenuated cardiac remodeling and atherosclerosis, increased STAT3 activity [57], decreased cardiac inflammation [58], oxidative stress and fibrosis [59], and apoptosis [60]. Although SGLT2 is not expressed by cardiomyocytes, empagliflozin directly inhibits myocardial sodium/proton exchanger-1, thus lowering cytoplasmic sodium and calcium levels and increasing mitochondrial calcium levels [61].

### Clinical benefit in HFpEF

The Empagliflozin Outcome Trial in Patients with Chronic Heart Failure with Preserved Ejection Fraction (EMPEROR-Preserved) trial provided the first clear demonstration of a prognostic benefit from empagliflozin in HFpEF [62]. This study randomized 5988 patients with NYHA class II–IV HF, EF > 40%, and NT-proBNP > 300 ng/L (or > 900 ng/L when in atrial fibrillation) to empagliflozin 10 mg once daily or placebo, in addition to usual therapy. About half of patients (49% in both groups) had diabetes, and two-thirds had EF ≥ 50%; 45% were women, and 50% had an estimated glomerular filtration rate (eGFR) < 60 mL/min/1.73 m^2^. Over 80% of patients in both groups were on ACEi/ARB, 2% on sacubitril/valsartan, about 86% on beta-blockers, and around 37% on mineralocorticoid receptor antagonists. Over a median 26-month follow-up, 13.8% of patients in the empagliflozin group and 17.1% in the placebo group experienced the primary endpoint of cardiovascular death or first HF hospitalization (HR 0.79 [0.69–0.90]; *p* < 0.001), with a number needed to treat of 31. Empagliflozin effect on the primary endpoint was consistent in patients with or without diabetes, was more evident in patients with EF < 50% than those with EF 50–59% (HR 0.71 [0.57–0.88] vs. HR 0.80 [0.64–0.99], and was not significant in patients with EF ≥ 60% (HR 0.87 [0.69–1.10]). Empagliflozin efficacy maybe therefore less evident for EF > 50%. Several other differences emerged (such as elderly vs. younger or obese vs. nonobese patients), but subgroup analyses were clearly underpowered. As for other endpoints, a 29% reduction in the risk of first HF hospitalization was remarked, with an early divergence of survival curves. The effect on cardiovascular death was neutral (HR 0.91 [0.76–1.09]), with a fair number of events (*n* = 463), suggesting a real lack of benefit. No effects emerged on all-cause death or all-cause hospitalization. Empagliflozin slowed down the yearly eGFR decline but did not improve a more clinically significant renal endpoint (i.e., profound and sustained decreases in eGFR or renal-replacement therapy) (HR 0.94 [0.73–1.24]), contrary to a 49% reduction in the empagliflozin outcome trial in patients with chronic HF and a reduced EF (EMPEROR-Reduced) trial (HR 0.51 [0.33–0.79]; *p* for interaction = 0.016) [63]. Empagliflozin had also a minor impact on the KCCQ clinical summary score. Empagliflozin had a satisfactory safety profile; uncomplicated genital and urinary tract infections and hypotension were reported more frequently with empagliflozin [62].

In a side-by-side comparison between the PARAGON-HF trial and the EMPEROR-Preserved trial [64], baseline characteristics were similar, including renal failure and diabetes prevalence, yet the former had higher EF (> 45% for eligibility, mean 57.5 ± 8.0%) than the latter (> 40% for eligibility, mean 54.3 ± 8.8%); moreover, in the PARAGON-HF, fewer patients were treated with beta-blockers (79.5%) and mineralocorticoid receptor antagonists (27.1%) than in the EMPEROR-Preserved trial (85.9% and 37.6%, respectively). Despite a higher statistical power in the PARAGON-HF due to a longer follow-up (median 35 months compared to 26 months in the EMPEROR-Preserved), the addition of neprilysin inhibition reduced the composite endpoint of cardiovascular death and total hospitalizations for HF only by 13% (rate ratio, 0.87 [0.75–1.01]; *p* = 0.059), while adding empagliflozin reduced the same end point by 21% (rate ratio, 0.79 [0.68–0.92]; *p* = 0.003). Neither sacubitril/valsartan nor empagliflozin exerted a significant effect on cardiovascular death [64]; moreover, at subgroup analysis, neither drug reduced the composite outcome in patients with EF > 57% or > 60%, respectively [14, 62]. In a further analysis of the EMPEROR-Preserved trial, empagliflozin reduced the combined risk of cardiovascular death, hospitalization for HF, or an emergency HF visit requiring intravenous treatment (HR 0.77 [0.67–0.87]; *p* < 0.0001) [1]. Empagliflozin reduced the total number of HF hospitalizations requiring intensive care (HR 0.71 [0.52–0.96]; *p* = 0.028) and the total number of hospitalizations requiring a vasopressor or positive inotropic drug (HR 0.73 [0.55–0.97]; *p* = 0.033). Fewer patients in the empagliflozin group reported outpatient intensification of diuretics (HR 0.76 [0.67–0.86]; *p* < 0.0001) compared to placebo. The benefit on total HF hospitalizations was similar in patients with an EF of 41–49% and 50–59% but was attenuated at higher EF values [1].

Overall, there is no definite answer to explain the reason for the observed clinical benefit of empagliflozin on HF hospitalizations in HFpEF patients, compared to all other drugs investigated so far. The most likely explanation relies on the combination of several cardiac and extracardiac mechanisms, including lower pulmonary and systemic congestion due to its diuretic effects, an improved cardiac energy production and microvascular function due to its metabolic effects, but also several other systemic effects including improved insulin sensitivity and fatty acid oxidation in the skeletal muscle, reduced systemic inflammation and oxidative stress, as well as some weight loss (− 1.2 kg [− 2.1 to − 0.3] compared to placebo). Extracardiac effects are indeed of utmost clinical benefit in HFpEF patients, where comorbidities are highly prevalent.

## Conclusions

Although some survival benefits have been reported with spironolactone and sacubitril/valsartan in patients with HFpEF, the magnitude of these effects has been modest, and the benefits have been apparent only in some patient subgroups. The EMPEROR-Preserved trial is a groundbreaking study showing for the first time a prognostic benefit in patients with HFpEF. Nonetheless, these positive results derived entirely from a reduction in the less clinically relevant endpoint, i.e., HF hospitalization. It will be interesting to see whether dapagliflozin reduces cardiovascular mortality in the dapagliflozin evaluation to improve the lives of patients with HFpEF (DELIVER) trial such that the results of the DELIVER and EMPEROR-Preserved trials would mirror the pattern of results of the dapagliflozin and prevention of adverse outcomes in HF (DAPA-HF) and EMPEROR-Reduced trials in patients with HFrEF. In the PRESERVED-HF trial, 324 HFpEF patients (with LVEF ≥ 45%, NYHA class II–IV symptoms, elevated natriuretic peptides, need for diuretic therapy, elevated filling pressures on cardiac catheterization or echocardiography) were randomized to dapagliflozin or placebo. Dapagliflozin improved Kansas City Cardiomyopathy Questionnaire Clinical Summary Score (KCCQ-CS) by 5.8 points (95% confidence interval (CI) 2.3–9.2, *p* = 0.001), 6-min walking distance by 20.1 m (95% CI 5.6–34.7, *p* = 0.007) and reduced body weight by 0.72 kg (95% CI 0.01–1.42, *p* = 0.046) at 12 weeks [65]. A meta-analysis on the effects of dapagliflozin and empagliflozin in HFpEF patients will be necessary to investigate the overall clinical effects of SGLT2i with higher statistical power and in clinically important subgroups, similarly to what has been done in HFrEF patients [66]. Further research is needed to explore potential benefits from tailoring the therapeutic approach to the individual phenotype (demographics, risk factors, comorbidities, underlying functional/structural cardiac abnormalities). Indeed, it is nearly impossible to achieve a complete standardization of HFpEF patient management, including their diagnostic, prognostic, and therapeutic algorithms based on universal cutoffs and treatment strategies. To this purpose, researchers are trying to unveil the heterogeneous nature of HFpEF through machine learning-based cluster analysis, so as to identify novel phenotypes (i.e., phenomapping) [67–70]. Phenomapping may improve HFpEF classification, conduct the design of future clinical trials, and lead to the development of novel targeted therapies [67]. More emphasis should be also placed on HFpEF patient-oriented outcomes, while the role of non-pharmacotherapeutic strategies, such as exercise training or caloric restriction, should be further investigated.

In summary, empagliflozin has emerged as a standardized therapy able to improve patient outcomes in HFpEF, although the survival benefit was driven by a reduction in HF hospitalization. The notion of tailoring the remaining therapy (including neurohormonal antagonists) according to the individual phenotype may deserve further consideration, especially if dapagliflozin is found to be no more effective than empagliflozin. Specific trials, possibly with a pragmatic design, should investigate treatment tailoring as an additional strategy to SGLT2 inhibition.

The table summarizes all clinical trials on neurohormonal drugs (beta-blockers, ACEi/ARBs, MRAs, ARNIs) to treat HFpEF, providing the main characteristics of patient populations, the endpoints and HR values with their 95% CI values. See text for further details. *6MWD*, 6-min walking distance; *ACEi*, angiotensin-converting enzyme inhibitors; *Aldo*-*DHF*, aldosterone receptor blockade in diastolic heart failure; *ARBs*, angiotensin receptor blockers; *ARNI*, angiotensin receptor neprilysin inhibitor; *CHARM-Preserved*, effects of candesartan in patients with chronic heart failure and preserved left-ventricular ejection fraction; *CIBIS*-*ELD*, Cardiac Insufficiency Bisoprolol Study in Elderly; *CV*, cardiovascular; *E/e′*, transmitral early diastolic velocity (*E*) over tissue Doppler early diastolic myocardial velocity (*E*′); *HF*, heart failure; *HFpEF*, heart failure with preserved ejection fraction; *HR*, hazard ratio; *I*-*Preserve*, Irbesartan in Heart Failure with Preserved Ejection Fraction Study; *LA*, left atrium; *LV*, left ventricular; *LVEF*, left ventricular ejection fraction; *MRA*, mineralocorticoid receptor antagonists; *NYHA*, New York Heart Association; *NS*, nonsignificant; *NT-proBNP*, N-terminal pro-B-type natriuretic peptide; *PARAGON*-*HF*, angiotensin receptor neprilysin inhibition in heart failure with preserved ejection fraction; *PARAMOUNT*, Prospective comparison of ARNI with ARB on Management Of heart failUre with preserved ejectioN fraction; *PEP*-*CHF*, perindopril in elderly people with chronic heart failure; *SENIORS*, Study of Effects of Nebivolol Intervention on Outcomes and Rehospitalization in Seniors; *TOPCAT*, Treatment of Preserved Cardiac Function Heart Failure With an Aldosterone Antagonist.

## Abbreviations

6MWD: 6-Min walking distance
ACC/AHA/HFSA: American College of Cardiology/American Heart Association/Heart Failure Association of America
ACEi: Angiotensin-converting enzyme inhibitors
AGEs: Advanced glycation end products
Aldo-DHF: Aldosterone Receptor Blockade in Diastolic HF
ARBs: Angiotensin receptor blockers
BADDHY: Safety and Efficacy of Bosentan in Patients With Diastolic Heart Failure and Secondary Pulmonary Hypertension
BNP: B-type natriuretic peptide
CHARM-Preserved: Chronic HF and preserved left-ventricular ejection fraction
ChI: Chronotropic incompetence
CIBIS-ELD: Cardiac Insufficiency Bisoprolol Study in Elderly
DAPA-HF: Dapagliflozin and Prevention of Adverse-Outcomes in Heart Failure
DD: Diastolic dysfunction
DELIVER: Dapagliflozin Evaluation to Improve the LIVEs of Patients With PReserved Ejection Fraction Heart Failure
ECV: Extracellular volume
EF: Ejection fraction
eGFR: Estimated glomerular filtration rate
EMPEROR-Preserved: Empagliflozin Outcome Trial in Patients with Chronic Heart Failure with Preserved Ejection Fraction
EMPEROR-Reduced: Empagliflozin Outcome Trial in Patients With Chronic Heart Failure and a Reduced Ejection Fraction
ESC: European Society of Cardiology
FAIR-HFpEF: Effect of IV Iron in Patients With Heart Failure With Preserved Ejection Fraction
HF: Heart failure
HFmrEF: Heart failure with midrange/mildly reduced ejection fraction
HFpEF: Heart failure with preserved ejection fraction
HFrEF: Heart failure with reduced ejection fraction
HR: Hazard ratio
I-Preserve: Irbesartan in patients with HF and preserved ejection fraction
INDIE-HFpEF: Inorganic Nitrite Delivery to Improve Exercise Capacity in Heart Failure with Preserved Ejection Fraction
IPF: Idiopathic pulmonary fibrosis
KCCQ: Kansas City Cardiomyopathy Questionnaire
KNO3CK OUT HFpEF: Effect of KNO3 Compared to KCl on Oxygen UpTake in Heart Failure With Preserved Ejection Fraction
LV: Left ventricular
MRAs: Mineralocorticoid receptor antagonists
NPs: Natriuretic peptides
NT-proBNP: N-terminal fraction of pro-BNP
NYHA: New York Heart Association
PAP: Pulmonary artery pressure
PARAGON-HF: Efficacy and Safety of LCZ696 Compared to Valsartan, on Morbidity and Mortality in Heart Failure Patients With Preserved Ejection Fraction
PARAMOUNT: Prospective Comparison of ARNI with ARB on Examination of Heart Failure with Preserved Ejection Fraction
PDE-5: Phosphodiesterase-5
PEP-CHF: Perindopril in elderly people with chronic HF
PH: Pulmonary hypertension
PIROUETTE: Pirfenidone for HFpEF, the Efficacy and Safety of Pirfenidone in Patients With Heart Failure and Preserved Left Ventricular Ejection Fraction
PREFER*-*HF: Effects of Iron Therapy in Heart Failure With Preserved Ejection Fraction and Iron Deficiency
QoL: Quality of life
RALI-DHF: Ranolazine for the Treatment of Diastolic Heart Failure
RAPID-HF: Rate-Adaptive Atrial Pacing In Diastolic Heart Failure
SENIORS: Study of the Effects of Nebivolol Intervention on Outcomes and Rehospitalization in Seniors with Heart Failure
SGLT2i: Sodium-glucose linked transporter-2 inhibitor
STEP-HFpEF: Semaglutide Treatment Effect in People with obesity and HFpEF
TOPCAT: Treatment of preserved cardiac function HF with an aldosterone antagonist
VO_2_: Peak oxygen consumption
